# Supplementary material for: Existence and uniqueness of neutral functional differential equations with sequential fractional operators
Source: PLoS One. 2024 Jul 16;19(7):e0304575. doi: 10.1371/journal.pone.0304575 (PMC11251599; doi:10.1371/journal.pone.0304575)
Supplement: Attachment — Submitted filename: Response to Reviewers.pdf [file pone.0304575.s001.pdf]

Revision of Manuscript ID: [PONE-D-24-09244]

EXISTENCE AND UNIQUENESS OF NEUTRAL FUNCTIONAL DIFFERENTIAL EQUATIONS WITH SEQUENTIAL FRACTIONAL OPERATORS

Response to Reviewers

Thank you for the opportunity to revise our manuscript,
" EXISTENCE AND UNIQUENESS OF NEUTRAL FUNCTIONAL DIFFERENTIAL EQUATIONS WITH SEQUENTIAL FRACTIONAL OPERATORS".
We would like to thank the reviewers for the careful review, and constructive comments and suggestions. Please see the colored text in the updated version of the manuscript for incorporated comments and suggestions.
Thank you for your consideration.

Sincerely,

Dr. Moumen, Ph.D.

We give below our responses to the reviewers' comments and suggestions.

Response to Reviewer 1

- **Comment 1:** Why generalized derivative considered? clarify it in introduction in last one paragraphs.

Response: *The generalized derivative is considered simply because we set G(t)=lnt, we obtain Caputo-Hadamard derivative , and when we take G(t)=t, we obtain Caputo fractional derivative.*

- **Comment 2:** Update the literature with recent work like:

1- On theoretical and numerical analysis of fractal–fractional non-linear hybrid differential equations."Nonlinear Engineering 13.1 (2024): 20220372.
2- Using a prior estimate method to investigate sequential hybrid fractional differential equations." Fractals 28.08 (2020): 2040004.
3- Application of topological degree method for solutions of coupled systems of multipoints boundary value problems of fractional order hybrid differential equations." Complexity 2017 (2017). Indian Journal of Pure and Applied Mathematics 51 (2020): 669-687.Indian Journal of Pure and Applied Mathematics 52.1 (2021): 27-38

Response: *We included the following necessary reference for our research:*

> *Ghazala Nazir, Kamal Shah, Thabet Abdeljawad, Hammad Khalil, Rahmat Ali Khan, Using a prior estimate method to investigate sequential hybrid fractional differential equations." Fractals 28.08 (2020): DOI: 10.1142/S0218348X20400046*

- **Comment 3**: Put some physical applications of hybrid problems.

Response*: The following paragraph has been added to the introduction*

"Hybrid problems, which involve the combination of different mathematical techniques or models to solve complex physical phenomena, have applications in various fields. Some examples of physical applications of hybrid problems include Fluid-structure interaction, electromagnetic-structural interaction, thermo-mechanical analysis, coupled heat and mass transfer, multiphase flow with chemical reactions, biomechanics".

Response to Reviewer 2

- **Comment 1:** What are the prospects of applying the G-Caputo operator derivatives fractional derivatives in practical applications?.

Response*: The application prospects of fractional derivatives, particularly those involving the G-Caputo operator, are quite promising across various fields. Here are a few areas where they can be particularly useful: Signal Processing, Control Systems, Finance and Economics, Materials Science, Biomedical Engineering, Electrochemistry, Mechanical Engineering.*

- **Comment 2:** Abstract should be re write.

Response*: Done*

- **Comment 3:** Clear explanations of classical solutions are expected.

Response*: Clear explanations are incorporated. See for example proof of Theorem 3.2.*

- **Comment 4:** Are the theoretical results in the paper dependent on the order of the fractional derivative? If they are independent of the order, then what is the significance of discussing such fractional derivative models?

Response*: Yes, the theoretical results depend on the order of the fractional derivative. See for example Lemma 1.5 and Theorem 2.2.*

• **Comment 5:** Compared to integer-order models or Riemann-Liouville/Caputo fractional-order models?

***Response**: The choice between integer-order models, Riemann-Liouville fractional-order models, and Caputo fractional-order models depends on the specific requirements of the problem at hand, including the nature of the system dynamics, the availability of initial conditions, and computational considerations. Each type of model has its own strengths and limitations, and researchers typically choose the most appropriate model based on the characteristics of the system being studied and the objectives of the analysis.*

• **Comment 6:** Authors can give an application for the problem. The authors are expected to provide numerical simulation or numerical example for the considered problem.

***Response**: Please see the example in Section 5.*

• **Comment 7:** No proper proof is provided in the example for proving all the Hypotheses are verified. How to verify those results?

***Response**: In the example, all the previously mentioned hypotheses were verified (lines 196 to 199).*

• **Comment 8:** To show the efficiency of the method, the comparison result with the existing method can be provided.

***Response**: We can present the comparison result with the current method in further.*

• **Comment 9:** One more example is expected to validate the theory part.

***Response**: More examples can be added, for example one can take two nonlinear function $\arcsin x $, $\arctan x$, respectively. However, due to the length of the paper, we only provided one example.*

• **Comment 10:** Update the bibliography with recent work relevant to the problems in the fractional calculus sense. For example, add the following paper that is closely related to this paper:

a) https://doi.org/10.1016/j.aej.2022.03.011

b) https://doi.org/10.1002/mma.9748

c) https://doi.org/10.1371/journal.pone.0297478

d) doi: 10.3934/math.2024403

e) https://doi.org/10.1016/j.rico.2024.100376

f) DOI:10.5890/DNC.2024.03.014

g) https://doi.org/10.1002/num.22652

Response: *We include some references that are necessary for our research as follows:*

c) https://doi.org/10.1371/journal.pone.0297478

d) doi: 10.3934/math.2024403

e) https://doi.org/10.1016/j.rico.2024.10037

We are very thankful to the reviewer for all his useful recommendations and guidelines.
